# Editorial: Creative approaches to appropriation and design: novel robotic systems for heterogeneous contexts

**DOI:** 10.3389/frobt.2024.1531132

**Published:** 2025-01-03

**Authors:** Richard Paluch, Felix Carros, Galina Volkova, Mohammad Obaid, Claudia Müller

**Affiliations:** ^1^ Information Systems, University of Siegen, Siegen, Germany; ^2^ Statistisches Bundesamt, Bonn, Germany; ^3^ Department of Computer Science and Engineering, Chalmers University of Technology and University of Gothenburg, Göteborg, Vastra Gotaland County, Sweden

**Keywords:** appropriation, care, nursing, social robot, human-robot interaction, humor, participatory design (PD), socio-informatics

The discourse on robotic systems in care is shifting. While the debates have focused on the varying degrees of use of robotic systems in care, and whether or how the work of carers can be substituted by robots (Dalton-Brown, 2020), the focus is now on the practical implementation of robotic systems in care (Mahmoudi Asl et al., 2022). Concerns among carers about job losses due to replacement have diminished, while the demographic trends in Europe contributing to the shortage of staff and the growing number of people expected to need care have been highlighted (European Commission, 2023). In the future, the focus will be more on how to establish adequate human-technology interaction in the care sector and on the appropriation of technology by care staff and other stakeholders from a human-centered perspective (Paluch et al., 2022).

Appropriation is a process in which technical artifacts are used and integrated into users’ specific contexts and practices, adapting them to their needs and reinterpreting their purpose beyond the initial design intentions. In addition, appropriation is a creative and dynamic process that is mediated by context and emerges in collaboration with others. The objective is not to dictate technological solutions but to engage users that are actively using the technology, with the aim of facilitating mutual learning about how users adapt to technology and shaping its design to be meaningful and relevant to their needs. The focus is on long-term use and the creative and playful appropriation of technologies. Thus, this process is about working democratically to explore how the technologies best fit the context. This should create anchor points in people’s lives that enable meaningful appropriation (Stevens and Pipek, 2018).

With regard to robots for care settings, a socio-informatics perspective is particularly interesting, as it provides sensitizing concepts that enable the investigation of such questions in different practical contexts (Wulf et al., 2015; Stevens et al., 2018). However, there is still a need to clarify the practicalities of using robots and how people in different care settings can appropriate them long-term (Carros et al., 2022; Paluch and Müller, 2022; Paluch et al., 2024). This question has been explored in several research projects, the findings of which are presented in this Research Topic. It became evident that a focus was placed on emotions in a multitude of research dimensions. Additionally, it was established that the entertainment value of a robot and its capacity to respond to humor are significant aspects (Oliveira et al., 2021).

Interaction with robots can be challenging due to their potential unfamiliarity. In care settings, it can be helpful that robotic systems are developed with a participatory design element and that the robots are adapted to the specific local needs (Carros et al., 2020; Carros et al., 2023). If caregivers and care recipients are taken seriously in the development process, robotic systems can be designed that are used creatively to assist and enhance the wellbeing of those in need of care. Only then, we believe, an appropriation of the robotic systems is possible, which would otherwise be constrained by rigid usage requirements.

Overall, we selected five papers for this Research Topic that take into account the multidisciplinary needs of developing such robotic systems and that observed the appropriation from the users of their devices:

The article by Graf et al. entitled “Distributed agency in HRI—an exploratory study of a narrative robot design” presents a plant watering robot. The study investigated how the robot’s agency is experienced in different contexts, how this affects the attribution of the robot’s behavior, and whether it increases the enjoyment of users. Appropriation processes were observed *in situ*, and particular attention was paid to people’s reactions. The examples relate to a university campus, focusing on younger users, and a nursing home where people with dementia are cared for.

The article “What helps, what hinders?—Focus group findings on barriers and facilitators for mobile service robot use in a psychosocial group therapy for people with dementia” by Wasic et al. discusses the use of robotic systems to support therapists in dementia therapy. An important aspect is the promotion of appropriation processes to support the use of robots. As part of the study, four focus groups were conducted over a period of 2 years to accompany the use of the *Scitos G5* robot. The focus groups generated suggestions for the use of the robot, which were then evaluated and assessed in therapy sessions. Ethical research topics were also discussed. A total of 13 applications were implemented in this way, which proved to be helpful for the therapeutic work. In addition to time, financial resources, or the certainty of expectations when using the robot, humor was also mentioned, especially with regard to ethical aspects. Jokes and humor are beneficial for human-robot interaction in therapy.

In their article “HoLLiECares - Development of a multi-functional robot for professional care”, Schneider et al. refer to a robot called *HoLLiE* that is used in two hospitals. Six of its functions are examined (1. Pushing wheelchairs; 2. Escorting patients to examination rooms; 3. Body movement instructions; 4. Documenting wounds; 5. Storing medication, and 6. Handling limp objects). In this context, the perspectives of carers and patients were included to assess the acquisition of the functions. By considering individual functionalities, it is easier to decide how to scale the use of robots appropriately. It becomes clearer when interaction with a carer is required and when robots can be used. The analysis covers a range of possible applications along a continuum from human interaction to robot-assisted support.

In their article “Nature redux: interrogating biomorphism and soft robot aesthetics through generative AI”, Christiansen et al. discuss the potential of generative AI. One focus is on the AI software used for image generation. Here, biologically inspired ideas for soft robotics are examined. One example is biomorphic aspects, which are said to have an optimizing effect on human-robot interaction. The inclusion of AI image generation techniques allows different stakeholders to participate in the design process, including those without design expertise. This can contribute to a democratization of robotic design and at the same time promote the reflection of different cultural views on the biomorphic aesthetics of robotic systems. This work is dedicated to the investigation of the limits and possibilities of AI image generation for creative processes in robot design. Furthermore, the results are analyzed in terms of how the design of soft robots can be mediated. This knowledge can be used for the participatory design of robotic systems.

Finally, in the article by Ushijima et al. “Predicting humor effectiveness of robots for human line cutting”, the authors discuss a security robot that prevents people from queue-jumping. The idea is that by telling jokes, the robot will react in such a way that people behave according to expectations and follow the rules. The authors began by creating a data set and developing a predictor of the effectiveness of humorous statements. They then simulated 13,000 situations in which people cut in line and collected 500 phrases via crowdsourcing that could be described as humorous. The most humorous phrases related to queue-jumping were systematically identified and compared with non-humorous phrases in video experiments. The video experiments simulated the situation to record viewers’ reactions. The humorous phrases proved to be more effective than the non-humorous phrases in preventing rule-breaking.

In conclusion, there is a strong case for looking closely at appropriation processes, namely, what happens when a robotic system is put into practice and what can be learned from this. An ethnographic approach offers the development team as well as caregivers and care recipients an additional perspective. The selected contributions focus on the possibilities that complex robotic systems open up for care, emphasizing aspects such as humor and democratization. From a praxeological standpoint, it is crucial to examine how these aspects manifest themselves in the respective situations and to draw conclusions regarding the design process. In the cases presented here, this applies both to the technical features of a robot and to how the different perspectives of the stakeholders can be integrated for collaboration.
